# Aptamer-based biosensors for the diagnosis of sepsis

**DOI:** 10.1186/s12951-021-00959-5

**Published:** 2021-07-19

**Authors:** Lubin Liu, Zeyu Han, Fei An, Xuening Gong, Chenguang Zhao, Weiping Zheng, Li Mei, Qihui Zhou

**Affiliations:** 1 Institute for Translational Medicine, Department of Stomatology, The Affiliated Hospital of Qingdao University, Qingdao University, Qingdao, 266003 China; 2 School of Stomatology, Qingdao University, Qingdao, 266003 China

**Keywords:** Aptamer-based biosensors, Nanomaterials, Diagnosis, Sepsis

## Abstract

Sepsis, the syndrome of infection complicated by acute organ dysfunction, is a serious and growing global problem, which not only leads to enormous economic losses but also becomes one of the leading causes of mortality in the intensive care unit. The detection of sepsis-related pathogens and biomarkers in the early stage plays a critical role in selecting appropriate antibiotics or other drugs, thereby preventing the emergence of dangerous phases and saving human lives. There are numerous demerits in conventional detection strategies, such as high cost, low efficiency, as well as lacking of sensitivity and selectivity. Recently, the aptamer-based biosensor is an emerging strategy for reasonable sepsis diagnosis because of its accessibility, rapidity, and stability. In this review, we first introduce the screening of suitable aptamer. Further, recent advances of aptamer-based biosensors in the detection of bacteria and biomarkers for the diagnosis of sepsis are summarized. Finally, the review proposes a brief forecast of challenges and future directions with highly promising aptamer-based biosensors.

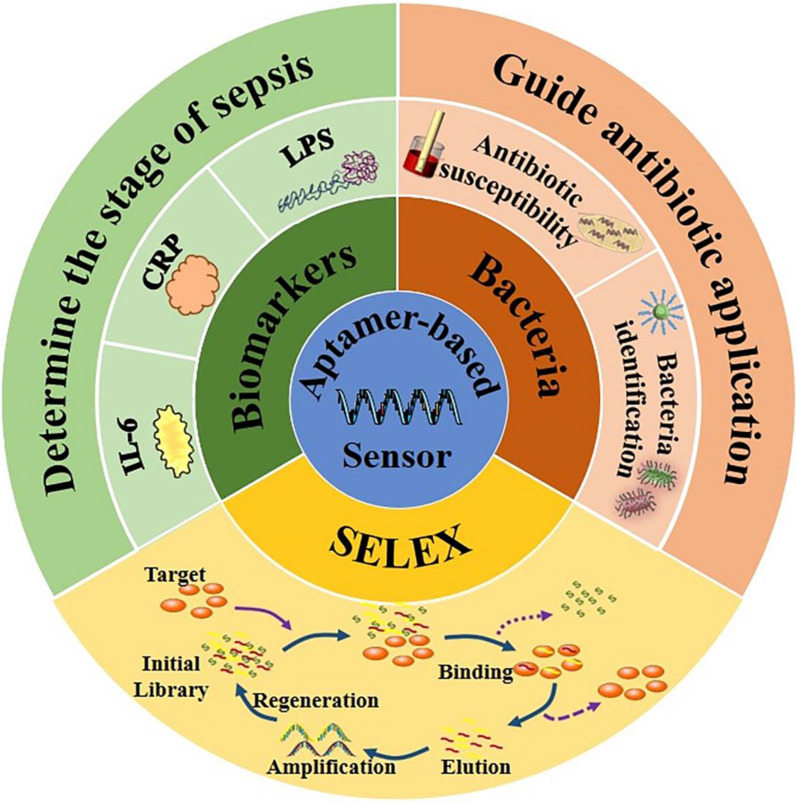

## Introduction

Sepsis, the syndrome of multiple organ dysfunction caused by immune disorders, is one of the most critical global issues in medicine due to the unacceptably high mortality rate [1–3]. Sepsis is an inflammatory disease mediated by the activation of the innate immune system which was induced by bacterial invasion either directly or indirectly [4, 5]. In particular, the most popular gram-positive isolates are *Staphylococcus aureus* (*S. aureus*) and *Streptococcus pneumoniae*. Meanwhile, *Escherichia coli* (*E. coli*), *Klebsiella*, and *Pseudomonas aeruginosa* dominate among gram-negative isolates [6, 7]. An epidemic international study of infection and sepsis containing more than 14,000 patients in 1265 participating intensive care units (ICUs) from 75 countries showed that 62% of the positive isolates were gram-negative organisms, 47% were gram-positive, and 19% were fungi [8]. The massive invasion of bacteria makes immunocytes activate and release kinds of cytokines. Some pathogenic components, such as lipopolysaccharide (LPS), can interact with the toll-like receptors (TLRs) of monocytes to activate transcription factor NF-kB which can promote the release of pro-inflammatory cytokines, such as tumor necrosis factor α (TNF-α) and interleukin 6 (IL-6) [9]. C-reactive protein (CRP), an acute-phase protein released by macrophages, remains the most frequently used biomarker for both infection and inflammation diagnosis in clinical practice [10].

According to a primary extrapolation of data from high-income countries, there are 19.4 million cases of severe sepsis annually around the world among 31.5 million cases of sepsis, with potentially 5.3 million death each year [11]. Although overall mortality decreases due to preventive measures, a more rapidly increasingly overall incidence rate of sepsis is revealed, demonstrating a continuing challenge [5]. However, the initial symptoms of sepsis are atypical and nonspecific, which is a clinical syndrome defined by a series of signs, symptoms, laboratory abnormalities, and characteristic pathophysiological derangements, resulting in a delayed diagnosis [5]. As research reported in *Critical Care Medicine*, over one-third of septic patients with atypical symptoms of infection are more likely to have a higher possibility to delay antibiotic administration and a higher risk of mortality [12]. The potential survival rate of sepsis falls dramatically up to 7.6% per hour without effective antibiotic treatment [13]. It is necessary to achieve the early diagnosis of sepsis to prevent the development of the disease. Sequential Sepsis-related Organ Failure Assessment (SOFA) score and bedside clinical score termed qSOFA (for quick SOFA) are recommended for early identification of sepsis [14]. Clinically, it is necessary to determine the species of pathogenic bacteria in time for the diagnosis of sepsis but conventional methods, such as blood cultures and molecular techniques, require multi-steps, resulting in time-consuming and demanding. They display low sensitivity which delays extremely the treatment of sepsis. The polymerase chain reaction (PCR), based on the detection of bacterial DNA, has the potential to reduce the diagnosis time to hours, but it still fails to detect the low-level blood infection [15–17]. Additionally, these methods are laboratory-based, and trained personnel for operation are needed a lot. In addition, blood biomarkers provide a valuable auxiliary role in the clinical status assessment of sepsis as the markers reflecting the severity of organism after infection and inflammation. Therefore, a rapid and sensitive method to diagnose sepsis in early stages is required urgently to ensure the rapid administration of appropriate antibiotics and prevent the occurrence of severe disease conditions, thereby saving human lives.

**Fig. 1:**
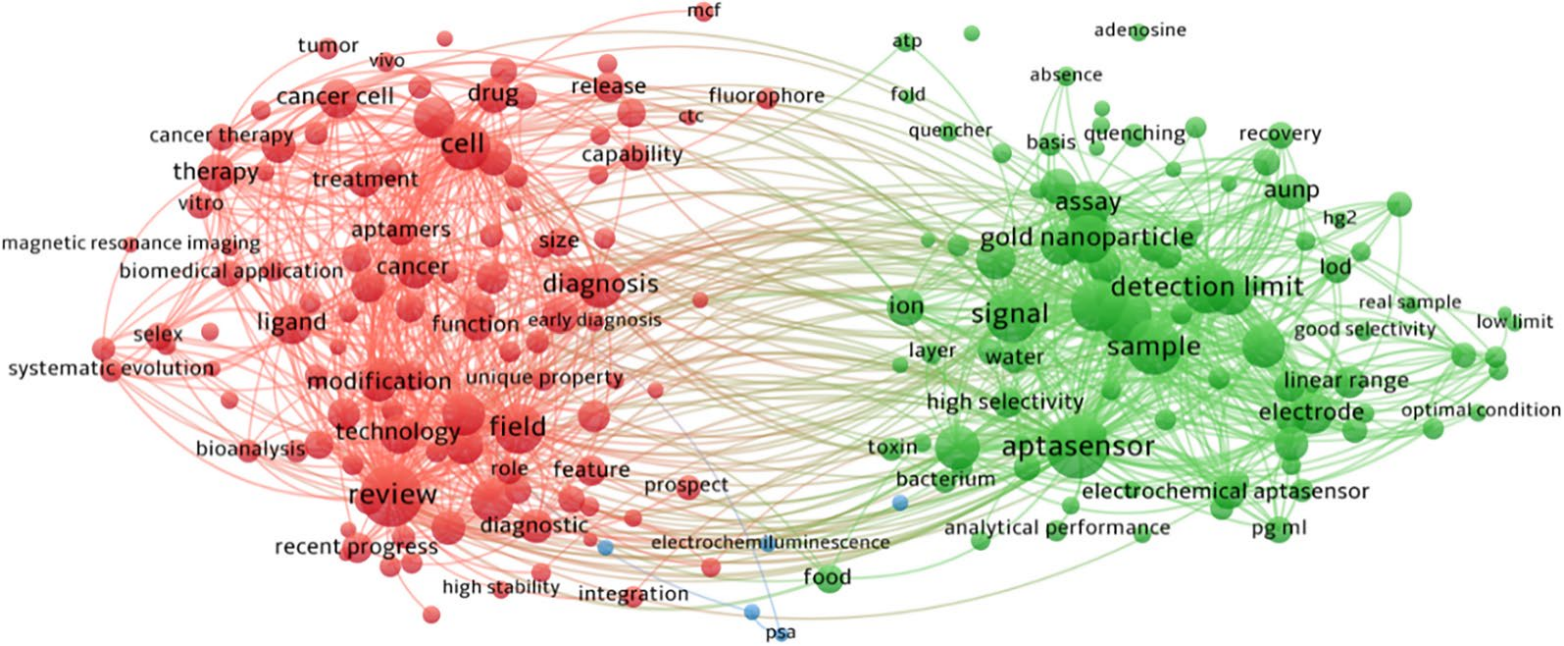
The analysis of keyword co-occurrences on aptamer and (nano)biointerface

Recently, the occurrence of aptamer-based sensors has attracted considerable attention for the diagnosis of sepsis owing to the dramatic efficiency for targets and the accuracy for detection [18]. Nucleic acid aptamers, identified by an *in-vitro* selection procedure called Systematic Evolution of Ligands by EXponential enrichment (SELEX), are single-stranded oligonucleotides (DNA or RNA) molecules that can bind to targets with high specificity and affinity [19, 20]. The aptamer is becoming increasingly popular nowadays because of the stability, easy accessibility, affordable prices, and minimal immune response compared with antibodies. Interestingly, a drug based on modified RNA aptamer, called Macugen (pegaptanib), has been approved by the Food and Drug Administration (FDA) for the treatment of age-related macular degeneration, showing the first successful commercial commodity [21]. Aptamers composed of nucleic acids can be modified easily by fluorescent dyes to achieve the detection visually [22]. In addition, aptamers are also used in homogenous assays which do not need to separate or wash because they bind to the target directly in a sequence-specific manner [23–25]. Recently, nucleic acid aptamers have been used widely as affinity receptors in combination with various signal transduction strategies based on nanomaterials in different kinds of biosensing platforms, including colorimetry, chemiluminometry, electrochemistry, fluorometry, and fluorescence anisotropy [26–31]. The aptamer-based biosensors have high detection sensitivity because aptamer can easily integrate with the signal amplification strategies, such as rolling circle amplification, CRISPR technology, PCR technology, LAMP technology, and magnetic separation technology. Xu et al. reported a dramatic increase in the sensitivity of bacteria detection through the combination of dual-functional aptamer and CRISPR-Cas12a assisted RCA [32]. Furthermore, the specificity of biosensing platforms comes from aptamer which can interact with the target by the unique structure transformation property of nucleic acid. The stability of aptamers has been improved significantly through the post-selection modification of aptamers and the direct selection of aptamers from libraries bearing modified backbones or nucleobases to ensure the stable functions of aptamer-based sensors [21, 33]. In addition, their inherent physicochemical characteristics of nanomaterials, including ultra-small size, high reactivity, and tunable surface modification, have enabled them to overcome some of the limitations and achieve the expected diagnostic and therapeutic effect [34–42]. The biosensors consist of (nano)biointerface and aptamer have been explored widely to detect bacteria and biomarkers, such as gold nanoparticles (NPs), graphene oxide, and carbon nanotubes, which play an indispensable role in improving the sensitivity and shortening the time in the detection for the target [22, 29, 30, 43]. The VOSviewer bibliometric visualization software was used to analyze co-occurrences on aptamer and (nano)biointerface (Fig. 1).

**Fig. 2:**
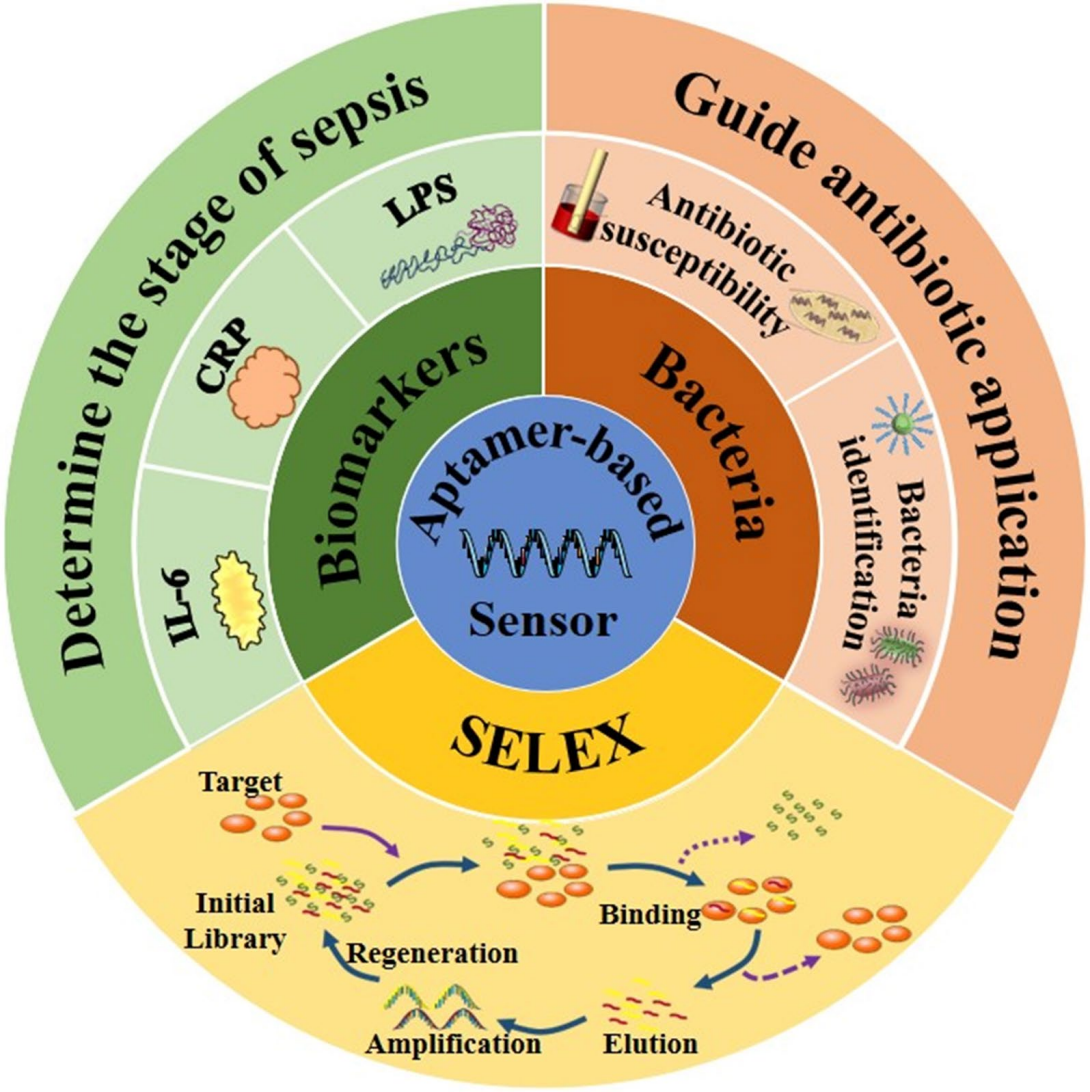
Aptamer-based biosensors in the detection of bacteria and biomarkers for the diagnosis of sepsis

In this review, the selection method of the nucleic acid aptamer is introduced briefly in the first. Also, recent advances of aptamer-based biosensors (Fig. 2) in the detection of bacteria and biomarkers (Table 1) for the diagnosis of sepsis are summarized. Finally, we summarize the mechanism and notable advantages or disadvantages of aptamer-based sensors in sepsis diagnosis (Table 2).

**Table 1:** Summary of aptamer-based detection of sepsis-related pathogens and biomarkers

Targets	Aptamer sequences	Nanomaterials	Sensor type/method	Type of aptamer	Length (nt)^a)^	Limit of detection (LOD)	References
*S. aureus*	I: 5’-TCC CTA CGG CGC TAA CCC CCC CAG TCC GTC CTC CCA GCC TCA CAC CGC CAC CGT GCT ACA AC-3’ II: 5’-TCC CTA CGG CGC TAA CCT CCC AAC CGC TCC ACC CTG CCT CCG CCT CGC CAC CGT GCT ACA AC-3’	AuNPs	Aptamer-conjugated GNPs and a resonance light-scattering detection system	DNA	40	–	[44]
	I: 5’-TCC CTA CGG CGC TAA CCC CCC CAG TCC GTC CTC CCA GCC TCA CAC CGC CAC CGT GCT ACA ACT TTT TTT TT-3’ II: 5’-TCC CTA CGG CGC TAA CCC CCC CAG TCC GTC CTC CCA GCC TCA CAC CGC CAC CGT GCT ACA ACT TTT TTT T-3’	Fe_3_O_4_@mTiO_2_	Capture platform based on Fe_3_O_4_@mTiO_2_ modified with target aptamer	DNA	71	10–2000 CFU/mL	[45]
	apt: 5’-GCA ATG GTA CGG TAC TTC CTC GGC ACG TTC TCA GTA GCG CTC GCT GGT CAT CCC ACA GCT ACG TCA AAA GTG CAC GCT ACT TTG CTA A-3’	–	Vertical capacitance apta-sensors	DNA	88	10^0^ CFU/mL Biofilm: 20% of the area	[46]
	apt: 5’-GCA ATG GTA CGG TAC TTC CTC GGC ACG TTC TCA GTA GCG CTC GCT GGT CAT CCC ACA GCT ACG TCA AAA GTG CAC GCT ACT TTG CTA A-3’	–	Electrical antimicrobial susceptibility test (e-AST) system	DNA	88	–	[47]
MASA	–	Streptavidin Magnetic Beads	CRISPR-Cas12a assisted RCA	–	–	10^2^–10^6^ CFU/mL	[32]
*E. coli*	apt: 5’-ATC CGT CAC ACC TGC TCT ACT GGC CGG CTC AGC ATG ACT AAG AAG GAA GTT ATG TGG TGT TGG CTC CCG TAT TTT TTT TTT-3’	Fe_3_O_4_@mTiO_2_	–	DNA	81		[45]
	apt: 5’-GCA ATG GTA CGG TAC TTC CCC ATG AGT GTT GTG AAA TGT TGG GAC ACT AGG TGG CAT AGA GCC GCA AAA GTG CAC GCT ACT TTG CTA A-3’	–	Vertical capacitance ap-tasensors	DNA	88	10^0^ CFU/mL Biofilm: 20% of the area	[46]
	apt: 5’-GCA ATG GTA CGG TAC TTC CCC ATG AGT GTT GTG AAA TGT TGG GAC ACT AGG TGG CAT AGA GCC GCA AAA GTG CAC GCT ACT TTG CTA A-3’	–	Electrical antimicrobial susceptibility test (e-AST) system	DNA	88	–	[47]
Peptidoglycan	I: 5’-TCG CGC GAG TCG TCT GGG GAC AGG GAG TGC GCT GCT CCC CCC GCA TCG TCC TCC C-3’ II: 5’-TCG CGC GAG TCG TCT GGG GGA CTA GAG GAC TTG TGC GGC CCC GCA TCG TCC TCC C-3’	–	–	DNA	I/II: 55	–	[48]
OMVs	I: 5’-ATA CCA GCT TAT TCA ATT GGG TGA GGG GGG GTT CAC AAC GTT AAA GAT AGA CGG GGG AAG ATA GTA AGT GCA ATC T-3’ II: 5’-ATA CCA GCT TAT TCA ATT CCG AGT CCA GAC TCA CCG CCG CCT CCT CAA GAC GTG CTG GAG ATA GTA AGT GCA ATC T-3’	–	Enzyme-linked aptamer assay	DNA	I/II: 76	*E. coli* DH5α: 0.13 ± 0.01 μg/mL *E. coli* K12: 3.70 ± 0.98 μg/mL *S.marcescens*: 0.23 ± 0.16 μg/m	[49]
*P.aeruginosa*	apt: 5’-CCC CCG TTG CTT TCG CTT TTC CTT TCG CTT TTG TTC GTT TCG TCC CTG CTT CCT TTC TTG-3’	–	Vertical capacitance aptasensors	DNA	60	10^0^ CFU/mL Biofilm: 20% of the area	[46]
	apt: 5’-CCC CCG TTG CTT TCG CTT TTC CTT TCG CTT TTG TTC GTT TCG TCC CTG CTT CCT TTC TTG-3’	–	Electrical antimicrobial susceptibility test (e-AST) system	DNA	60	–	[47]
*K.pneumoniae*	apt: 5’-GCA ATG GTA CGG TAC TTC C(N45)-CAA AAG TGC ACG CTA CTT TGC TAA-3’	–	Electrical antimicrobial susceptibility test (e-AST) system	DNA	44	–	[47]
*E. faecalis*	apt: 5’-ATC CAG AGT GAC GCA GCA CGA CAC GTT AGG TTG GTT AGG TTG GTT AGT TTC TTG TGG ACA CGG TGG CTT A-3’	–	Electrical antimicrobial susceptibility test (e-AST) system	DNA	70	–	[47]
LPS	apt: 5’ -CTT CTG CCC GCC TCC TTC C-(45 N)-GGA GAC GAG ATA GGC GGA CAC T-3’	Gold disk electrodes	Electrochemical	DNA	86	0.01–1 ng/mL	[50]
		Gra AuNPs	Electrochemical			8.7 fg/mL 10–50 fg/mL	[51]
		Gold atomic cluster	Electrochemical			7.94 × 10^–21^ M and 0.01aM–1 pM	[52]
		RGO/AuNPs	Electrochemical			1 fg/mL	[53]
		RGO/AuNPs	Electrochemical			0.2 fg/mLand 0.001–0.01 pg/mL	[54]
		MoS_2_ AuNPs RGO	Voltammetric biosensor			3.01 × 10^–5^ ng/mL and 5.0 × 10^−5^ ng/mL to 2.0 × 10^–2^ ng/mL	[55]
		–	Optical sensor			5.5 pg/mL– 100 ng/mL	[56]
		SLG	Acoustic wave biosensor			3.53 ng/mL 0–100 ng/mL	[57]
		GO	Fluorescence quenching efficiency			15.7 ng/mL and 25–1600 ng/mL	[58]
		RGO	Fluorescence quenching efficiency Continuous Injection-Electrostacking			8.3 fM	[59]
IL-6	Model number: ATW0082 ATW0077	AuNPs	Optical approach	–	–	1.95 μg/mL	[60]
	apt: 5′-GTC TCT GTG TGC GCC AGA GAC ACT GGG GCA GAT ATG GGC CAG CAC AGA ATG AGG CCC-3′	AuNPs	Electrochemical	–	–	1.6 pg/ml	[61]
	Model number: ATW0077	Carbon nanotube	Microfluidic-based approach	–	–	1 pg/mL–10 ng/mL	[62]
	apt: 5’- GGT GGC AGG AGG ACT ATT TAT TTG CTT TTC T -3’	GR	Field-Effect Transistor-Based Approach	–	–	139 fM	[63]
	–	GR	Field-Effect Transistor-Based Approach	–	–	618 fM	[64]
CRP	apt: 5’-GGC AGG AAG ACA AAC ACG ATG GGG GGG TAT GAT TTG ATG TGG TTG TTG CAT GAT CGT GGT CTG TGG TGC TGT-3’	–	Optical fiber sensor	DNA	72	2–20 mg/mL	[65]
	apt: 5’-GCC UGU AAG GUG GUC GGU GUG GCG AGU GUG UUA GGA GAG AUU GC-3’	–	Luminex xMAP technology	RNA	44	0.4 mg/L	[66]
	apt: 5’-CGA AGG GGA TTC GAG GGG TGA TTG CGT GCT CCA TTT GGT G- 3’	AuNPs	Optical nanosensor	DNA	40	1.77 pM	[67]

**Table 2:** The mechanism and notable advantages or disadvantages of aptamer-based biosensors

Sensor Type/Method	Mechanism of Action	Comments	References
Aptamer-conjugated GNPs and a resonance light-scattering detection system	Aptamers are combined onto GNPs followed by bead-based amplification, one bacterial cell was capable of generating more than 10^4^ GNPs after amplification, and amplified GNPs could be detected by the light-scattering–sensing system	Very short detection time. The detection of a single cell can be reached within 1.5 h without complicated equipment such as thermal cyclers or centrifuges	[44]
CRISPR-Cas12a assisted RCA	The specificity based on the dual functionalized aptamers can initiate bioconjugation to specifically recognize the protein targets and can also convert the protein recognition to nucleic acid signals	Accurate identification and high-sensitive detection of MRSA. Dual amplification of the nucleic acid signal	[32]
Capture platform based on Fe_3_O_4_@mTiO_2_ modified with target aptamer	First, the complex was incubated with blood samples and the aptamer would connect with the target bacteria. After that, the bacteria captured by Apt-Fe_3_O_4_@mTiO_2_ NPs were concentrated with the help of the magnetic field	High bacterial-captured efficiency of about 80%, short detection time within 2 h, and little cross-react with the nontarget components in blood	[45]
Fe_3_O_4_-Ce6-Apt nanosystem	Simultaneous blood bacterial species identification and enrichment can be achieved in a single step, and then, enriched bacteria can be detected with fluorescence microscopic determination	Identify and enrich the bacteria in the sepsis blood sample for 1 h. Blood disinfection	[68]
Enzyme-linked aptamer assay	Construct ELAA	High sensitivity to bacterial OMVs as low as 25 ng/mL. A new possibility for the development of cell-free bacterial sensors using bacterial OMVs instead of living bacterial cells	[49]
Vertical capacitance aptasensors	Some bacteria, culture in blood culture media comprising blood (0.2 mL) and culture media (0.8 mL), the biofilm formation and bacterial growth could be detected by measuring capacitance changes at f = 0.5 and 10 kHz, respectively. After treated with antibiotics, the sensitivity of bacteria to antibiotics can be judged by this change	Short AST time within 12 h	[46]
e-AST system	The e-AST system is composed of 60 aptamer-functionalized capacitance sensors, of which 2 sensors were used for the negative control, 3 sensors for positive control, and other 55 sensors for the determination of antibiotic sensitivity to 11 antibiotics at 5 different concentrations	Short AST time within 6 h	[47]
Voltammetric biosensor	After using aptamers immobilized by RGO, and MoS_2_ is also applied as the matrix of the biosensor with the application of RGO and AuNPs	Simplified operation sequence with fast response and high recovery rate. PEI-rGO-MoS_2_ nanocomposite with a larger specific surface area, thermal stability and electrical conductivity increases the sensitivity of the sensor	[55]
Acoustic wave biosensor	The SLG film first connects with CS, and then the amino groups in the CS react with the aldehyde in GA to form C = N bonds. After that, the aldehydes groups in GA react with the amine-functionalized aptamer, which is ready for the specific detection of endotoxin	Rapid, simple operations and low costs. Excellent stability from the air phase to the liquid phase	[57]
Microfluidic-based approach	Real-time response of the sensor conductance is monitored with increasing concentration of IL-6, exposure to the sensing surface in buffer solution, and clinically relevant spiked blood samples	Sensitive detection of IL-6 at low concentrations	[62]
Luminex xMAP technology	xMAP assays typically employ a sandwich-type format using antibodies for the capture. For this assay, an RNA aptamer that binds CRP is conjugated to beads to act as the capture agent	The number and type of analytes by using aptamers alone or in conjunction with antibodies expand and the use of sample volumes is low	[66]
Optical sensor	The signal output mode is an optical image, small changes can be converted into optical signals for output	Compatibility to a wide range of surface modifications. The detection limit of the sensor slightly changed with increased use. Some cross-reactivity towards the unspiked human serum	[56, 60, 65, 67]
Fluorescence quenching efficiency	The concentration of LPS can be quantitatively analyzed by observing fluorescence changes	Little consumption of sample. Low recovery of serum sample	[58, 59]
Field-Effect Transistor-Based Approach	The graphene surface immobilized aptamer is unfolded without IL-6 and it would fold after binding with the target. These aptamer structural changes bring the negative charges in IL-6 to the proximity of the graphene-liquid interface	Low-voltage operation (< 1 V), inherent gain amplification, biocompatibility and miniaturization	[63, 64]
Electrochemical	Electrochemical sensors are constructed using various nanomaterials	Gold disk electrodes: Short detection time and little cross-interaction reactivity to plasmid DNA, RNA, proteins, saccharides, and/or lipids which are most likely to coexist with LPS assay Gra AuNPs: Overcome the disadvantage of limited nicking endonuclease recognition and integrate molecular biological technology and nano-biotechnology with electrochemical detection to cascade signal amplification, which can detect target LPS down to the femtogram level Gold atomic cluster: Simple sensor fabrication compared with other electrochemical sensors for LPS RGO/AuNPs: Short LPS detection time within 35 min. Enhanced electrode performance and low LOD down to femtomolar level AuNPs: Label-free detection, simple experimental protocol, high selectivity and low limit of detection	[50–54, 61]

## The SELEX of aptamer

**Fig. 3:**
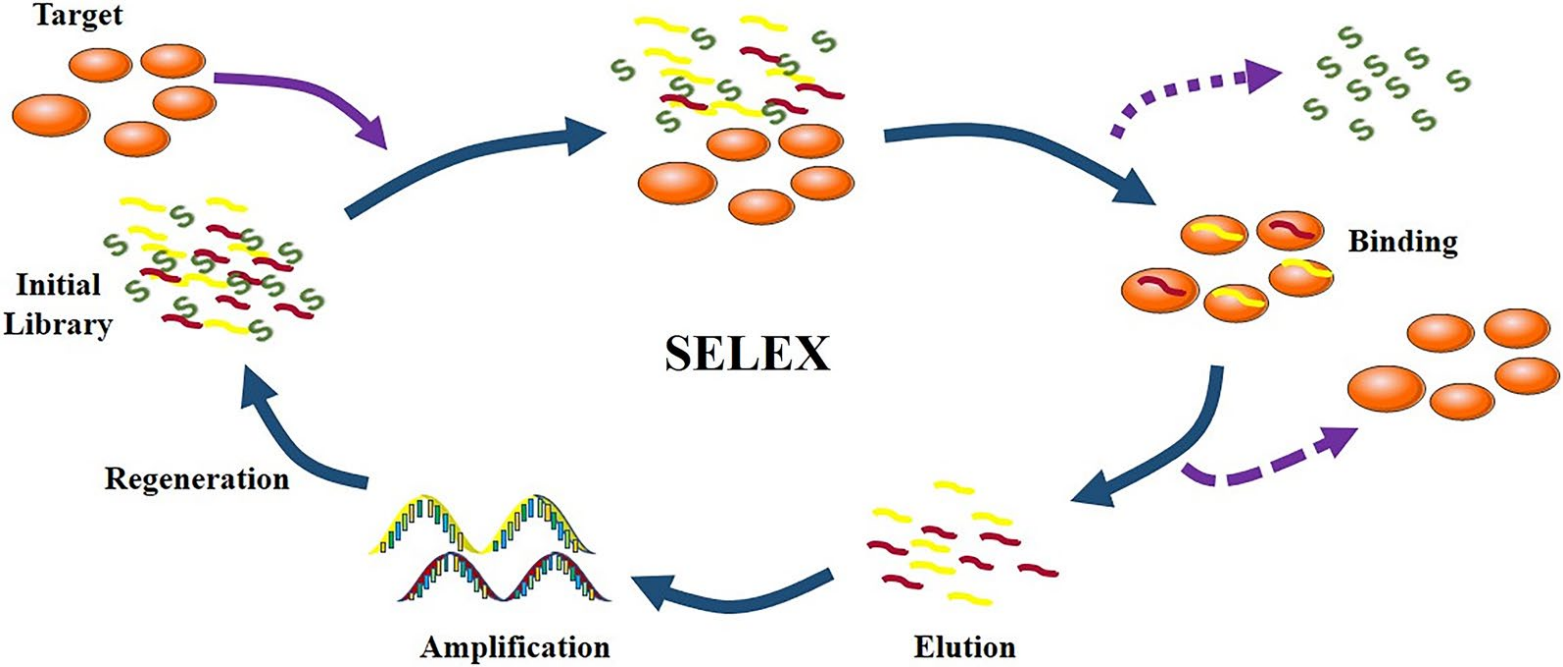
The process of SELEX

Nowadays, the selection of aptamers is on the basis of systematic evolution of ligands by SELEX which is a gold-standard strategy that can select specific and sensitive aptamers from random single-stranded nucleic acid sequence library [69]. In 1990, Ellington and Szostak successfully screened out oligonucleotides and named the “aptamer”, which is the first time to get aptamer from RNA library through this method [19, 20]. Briefly, there are three steps included in the selection cycle for DNA [70]. First of all, targets are incubated with the library containing randomized sequences, obtaining a complex of target and sequences. Secondly, nonspecific aptamers and the targets are separated respectively, and bound sequences are preserved. Finally, the proper sequences will be amplified through PCR. The selection cycle is then repeated until the sequence of the desired affinity is enriched. In every selection round, more affiliative aptamers are selected (Fig. 3). There are tiny differences of the SELEX for RNA aptamers that RNA should reverse transcription into DNA first and homologous DNA is transcribed into RNA after the selection.

However, there are some demerits in the traditional methods, such as time-consuming and huge costs [71]. Nowadays, some new methods based on traditional SELEX have been developed to overcome shortcomings, like cell SELEX, magnetic bead-based SELEX, in vivo SELEX, in Silico SELEX and so on, which aims to save time, use expediently and raise efficiency [69, 70, 72].

## Aptamer-based detection of pathogenic bacteria

Sepsis, a serious infection syndrome that can cause tissue damage and multisystem organ failure, is usually due to the presence of pathogenic bacteria in the bloodstream, resulting in high mortality in the world [73]. Importantly, sepsis has a high mortality rate due to the inability to quickly identify pathogens in the early stages of infection. Conventionally, blood culture, called the “gold standard”, is mostly used for bacteria detection in clinical, hence time-consuming, costly, and trained personnel for the operation needed in great demand.

Therefore, the rapid, accurate, and easy detection of bacteria is required urgently for the early diagnosis and therapy of sepsis. Aptamer-based sensors have a great potential to solve this problem because of sensitivity, specificity, and rapidity. Here, we discuss some articles about the detection of sepsis-related bacteria through aptamer-based sensors.

### Aptamer-based detection of a single type of pathogenic bacteria

**Fig. 4:**
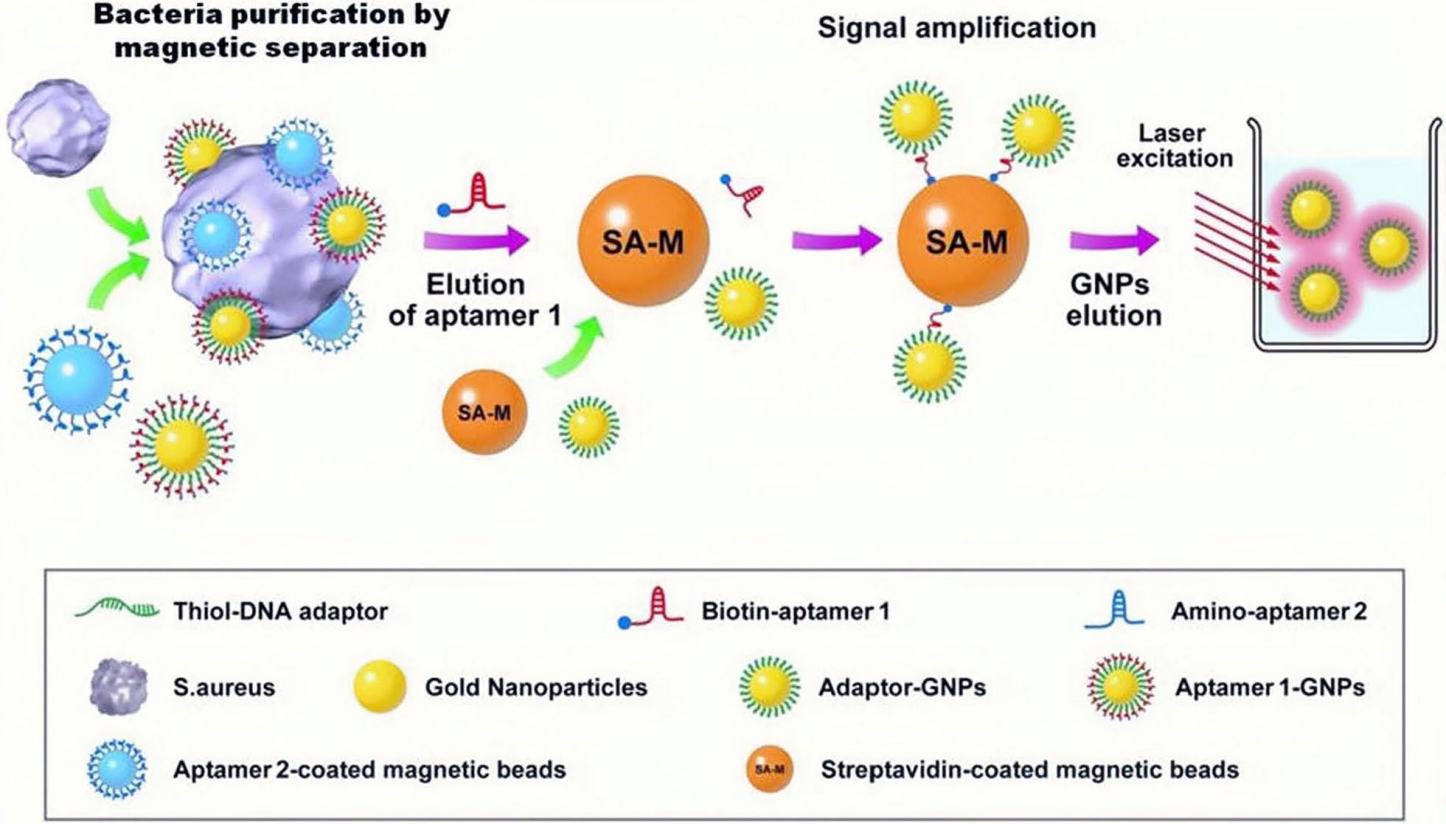
Bead-based amplification in the detection of unbound *S. aureus* using aptamer-conjugated GNPs [44]

This section pays attention to sensors designed for detecting pathogenic whole cells that can be targeted by aptamers. The first example of aptamer-based sensors that will be introduced was created to detect *S. aureus*, a common pathogen of sepsis [44, 74]. First of all, SA17 and SA61, two DNA aptamers that showing high specificity and nanomolar affinity with *S. aureus*, were modified on magnetic beads and gold nanoparticles (GNPs) separately. After that, quantitative PCR (qPCR) was used to quantify the number of aptamers or aptamer-conjugated GNPs linked with single *S. aureus* cells. To improve the sensitivity of detection to *S. aureus*, aptamers were attached with NPs followed by amplification based on magnetic beads (Fig. 4). Using this ingenious way, a single *S. aureus* cell could be detected within 1.5 h without expensive equipment.

In another study, Xu et al. realized the detection for *Methicillin-Resistant Staphylococcus aureus* (MRSA) using dual-functional aptamer and CRISPR-Cas12a assisted RCA[32]. Different from the recognition of the whole cell, the aptamer recognizes MRSA depending on the penicillin-binding proteins2a (PBP2a), which shows a low affinity for β-lactam antibiotics. The biosensing process could be divided into two steps: bacteria isolation based on the protein A aptamer (Apt A) and signal amplification. Protein A is a membrane protein shared by both MRSA and *S. aureus*. In the first step, Streptavidin Magnetic Beads (SMBs) were mixed with Apt A to get the capture complex (SMBs-Apt A), and the protein A positive bacteria could be enriched by the SMBs-Apt A complex via the aptamer-protein interaction for the next step. The second aptamer (Apt B) was made of PBP2a specific aptamer and a Blocker. The Blocker was released from Apt B to touch off the following RCA when the Apt B connected with PBP2a on the surface of MRSA. Via the integration of attached RCA and CRISPR-Cas12a assisted trans-cleavage, dual amplification of the nucleic acid signal was obtained. Furthermore, the output above was consistent with the traditional colony count in the four groups of serum samples, which proved the feasibility of this method for clinical sample detection.

**Fig. 5:**
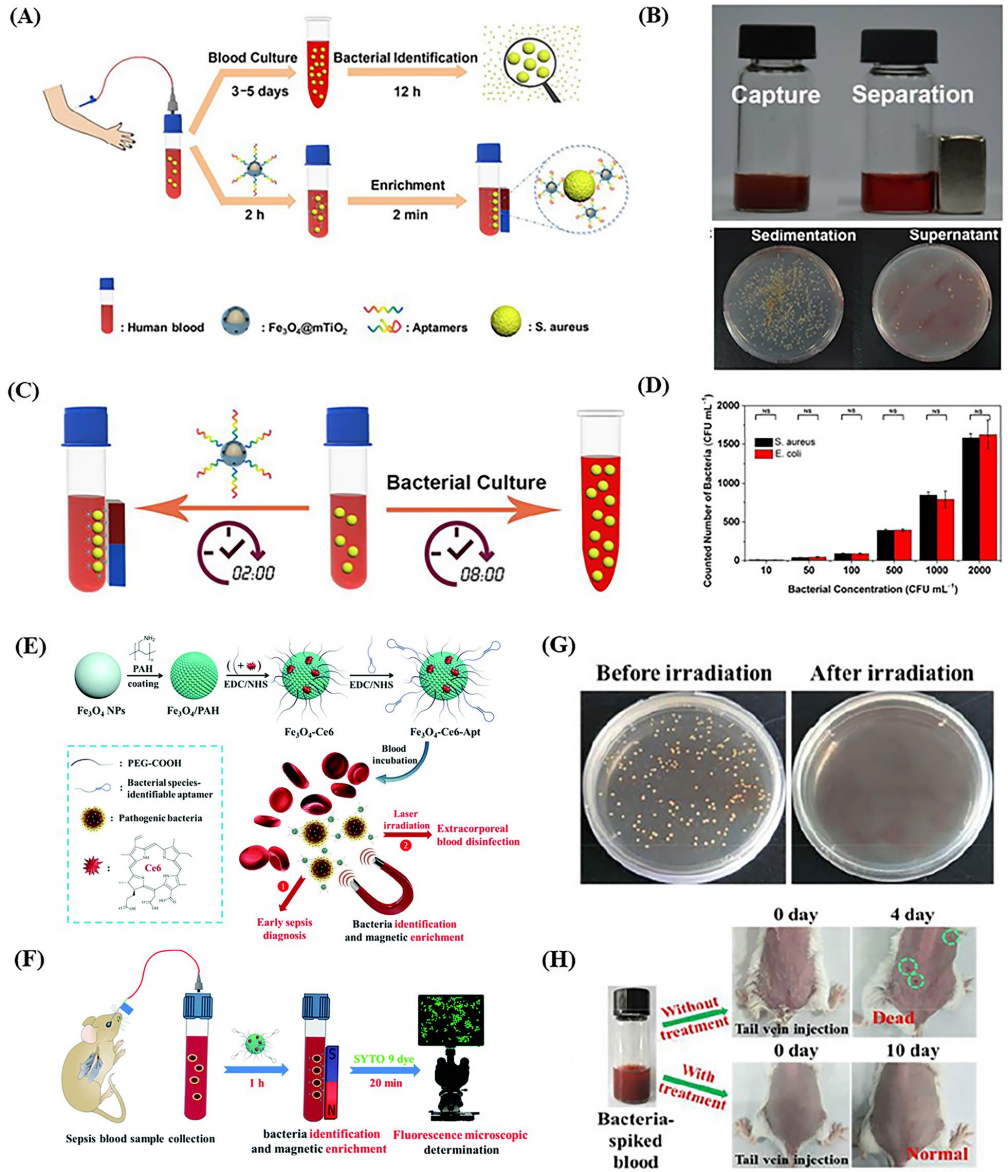
The Apt-Fe_3_O_4_@mTiO_2_ nanosensor (**A**–**D**). **A** Conceptual strategies to enrich and identify pathogenic bacteria in human blood. Top: conventional blood culture. Down: the aptamer-based capture platform. **B** Photographs and agar plates showing the bacteria capture with and without a bar magnet. **C** Schematic representation of detection time for enriching and identifying pathogen in human blood samples based on that aptamer-based capture platform (left) and conventional blood culture (right). **D** Bacteria counted numbers enriched by Apt-Fe_3_O_4_@mTiO_2_ nanosensor at a low concentration range (10–2000 CFU/mL) [45]. The Fe_3_O_4_-Ce6-Apt nanosystem (**E–H**). **E** Schematic illustration of strategies for early sepsis diagnosis and extracorporeal blood disinfection based on Fe_3_O_4_-Ce6-Apt nanosystem. **F** Illustration of the process of Fe_3_O_4_-Ce6-Apt nanosystem-based strategy for the bacterial enrichment and identification within 1.5 h. **G** Agar plate photographs for live bacterial units. The blood samples containing *S. aureus* (10^6^ CFU) were incubated with Fe_3_O_4_-Ce6-Apt nanosystem before and after NIR laser irradiation for 5 min. **H** Photographs of the mice transfused with the blood samples containing *S. aureus* (10^6^ CFU) with and without disinfection treatment at different times [68]

There are some studies that specific target aptamers and magnetic NPs were used together to identify and collect pathogenic bacteria in blood samples with low bacterial concentrations and achieved the rapid qualitative or quantitative detection of bacteria. Shen et al. created a capture platform that consisted of a mesoporous TiO_2_ coated magnetic NP and modified with target aptamer (Apt-Fe_3_O_4_@mTiO_2_) to reduce the time of detection [45]. First, the complex was incubated with blood samples and the aptamer would connect with target bacteria by folding into the sequence-defined unique structure when it was exposed to bacteria. After that, the bacteria captured by Apt-Fe_3_O_4_@mTiO_2_ NPs were concentrated with the help of the magnetic field to recognize pathogenic bacteria (Fig. 5A). Compared with the control group, the number of *S. aureus* decreased markedly in the supernatant after captured by a bar magnet within 2 min (Fig. 5B). Meanwhile, the Apt-Fe_3_O_4_@mTiO_2_ nanosensor had a higher efficiency (2 h) than conventional blood culture (8 h) to capture bacteria when 10^4^ CFU/mL bacteria were spiked into blood (Fig. 5C). To verify the reliability of the capture platform at low bacterial concentrations, two representative bacteria, *S. aureus* and *E. coli*, were used as model bacteria and the results showed that bacterial capture was up to 80% (10–2000 CFU/mL) (Fig. 5D). In addition to bacterial capture for diagnosis, Wang et al. accomplished efficient extracorporeal blood disinfection taking advantage of magnetic NPs functionalized with chlorin e6 molecules and bacterial species-identifiable aptamers (Fe_3_O_4_-Ce6-Apt) (Fig. 5E) [68]. Fe_3_O_4_-Ce6-Apt nanosystem could identify and enrich the bacteria through incubated with sepsis blood sample for 1 h and the enriched bacteria were imaged by fluorescence microscopy to quantitatively evaluate the number (Fig. 5F). After near-infrared (NIR) laser irradiation for 5 min (660 nm, 0.8 W/cm^2^), the agar plate showed a few bacteria (Fig. 5G). Through the conditions of the mice, the treatment for sepsis therapy based on blood disinfection by Fe_3_O_4_-Ce6-Apt was evaluated preliminarily (Fig. 5H). Although this aptamer-based sensor has shown advantages in the detection and therapy of sepsis, there is still much unknown about long-term safety in the human body.

### Aptamer-based detection of several types of pathogenic bacteria

In clinical practice, sepsis is commonly caused by the infection of a variety of bacteria, therefore, some scholars have constructed aptamers aiming at common components of bacteria, such as peptidoglycan and membrane vesicles [48, 49]. Compared with detecting one type of bacteria, these kinds of aptamers can simultaneously detect the existence of multiple types of sepsis-related bacteria, enabling patients to be diagnosed in an early and timely manner, guiding the use of antibiotics and reducing the mortality rate of patients.

Peptidoglycan is a kind of cell wall polymer shared by both gram-positive and gram-negative bacteria, which plays a critical role in the survival of bacteria and is closely related to the pathogenicity of bacteria [75, 76]. Ana et al*.* developed adapters that could recognize bacterial peptidoglycans, called Antibac1 (AT1) and Antibac2 (AT2), and found that both AT1 and AT2 have a high affinity for *E. coli* and *S. aureus* [77]. In subsequent work, they went on to explore the ability of AT1 and AT2 to bind to causative agents of bacterial-borne sepsis [48]. The results showed that these aptamers bound with high efficiency to the main agents of bacterial sepsis, including four gram-positive and seven gram-negative bacterial, and the affinity of AT1 and AT2 to bacteria was assessed by real-time quantitative PCR. This work demonstrated that ssDNA aptamers targeting bacterial peptidoglycan can recognize multiple types of septic pathogens and can be used to develop universal biosensor probes, which is of great significance for the rapid and sensitive detection of sepsis in clinical practice.

**Fig. 6:**
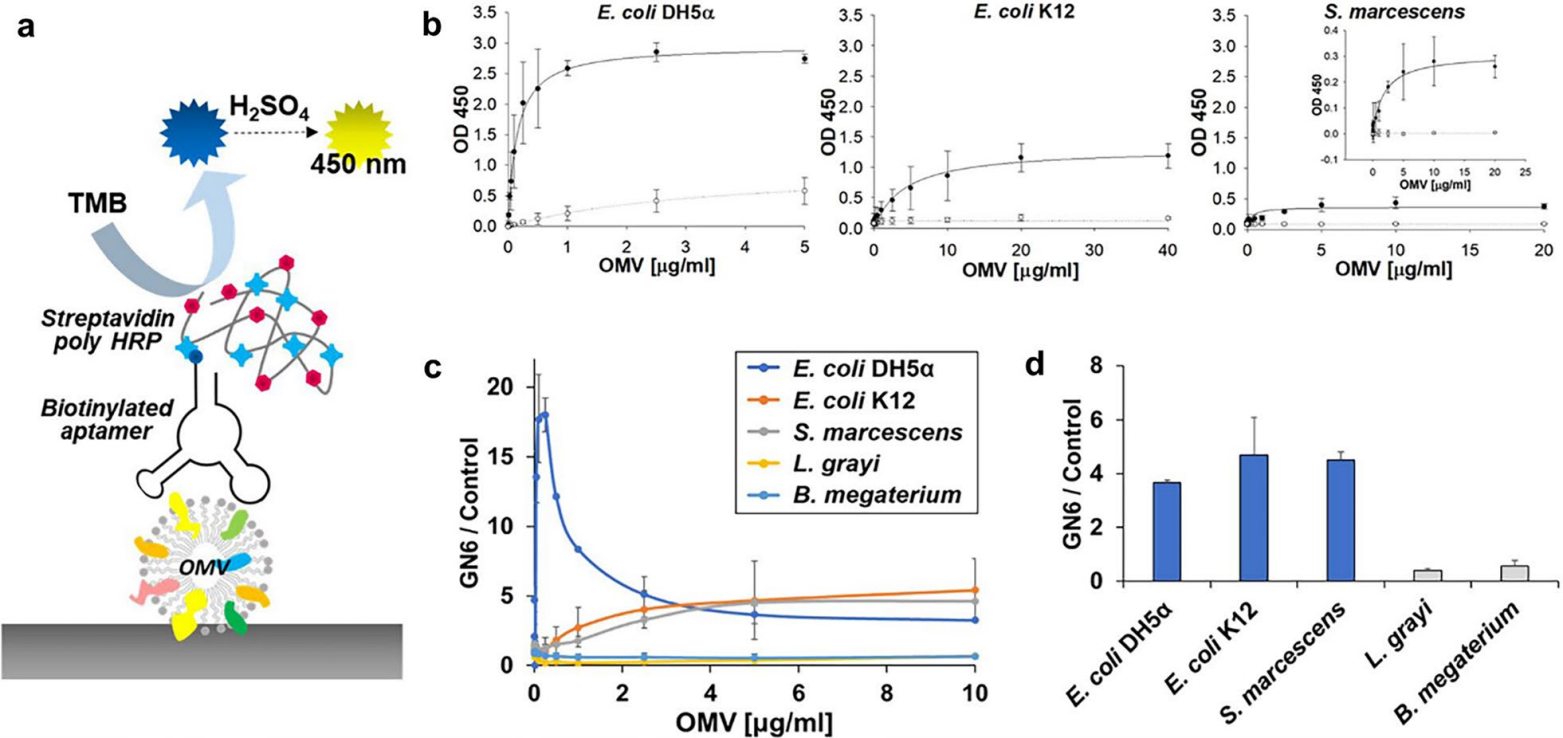
The scheme of GN6 ELAA and the specificity of GN6 to some OMVs [49]

Gram-negative bacteria are the main pathogenic bacteria leading to clinical sepsis. Their outer membrane produces and secretes outer membrane vesicles (OMVs) that can carry several virulence biomolecules and endotoxins [78]. OMVs are known to be important pathogenic agents that improve bacterial survival and trigger immune responses in host cells [79]. Therefore, Shin et al. developed a kind of broadly cross-reactive aptamers for the OMVs from gram-negative bacteria and built an Enzyme-linked aptamer assay (ELAA) (Fig. 6) [49]. The results showed that ELAA successfully detected OMVs from a variety of gram-negative bacteria, which provides a new possibility for the development of cell-free bacterial sensors using bacterial OMVs instead of living bacterial cells. Detection of live bacteria in the blood is difficult due to the immunomodulatory nature of host cells and the bactericidal activity of serum, suggesting that detecting bacterial OMVs in clinical blood samples may be more effective than detecting bacterial cells.

### Real-time monitoring of bacterial growth and AST

**Fig. 7:**
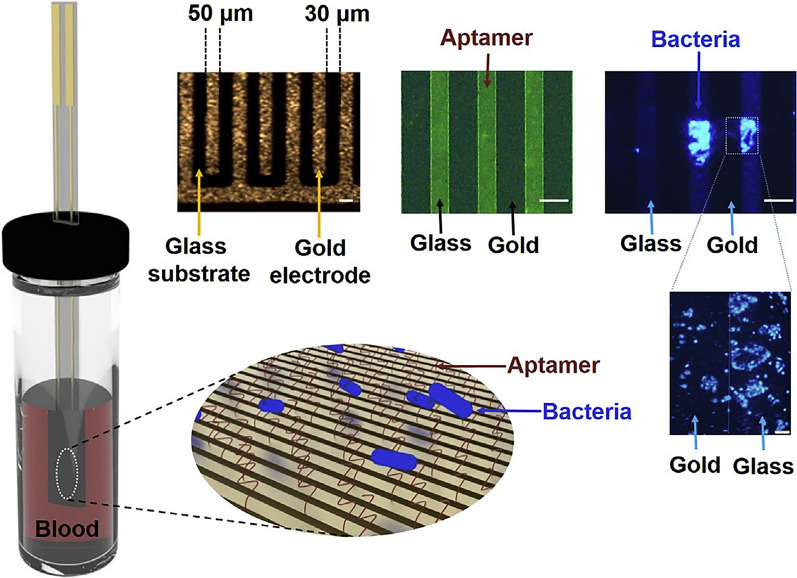
Scheme of vertical capacitance aptamer-functionalized sensor [46]

It is well-known that the survival rate of sepsis falls by an average of 7.6% for every hour of delay ineffective antibiotic treatment [13]. Patients’ survival could be highly improved if they received timely and effective antibiotic treatment, but antimicrobial susceptibility test (AST) results usually require more than three days with conventional methods [80–82]. In addition to the quantitative and qualitative detection of bacteria, the aptamer sensor can monitor the real-time growth of bacteria and biofilm, which continuously and timely evaluate the internal infections and the effectiveness of antibiotics [46, 47]. For instance, Song et al. developed a vertical capacitance aptamer-functionalized sensor (Fig. 7) that significantly shorten the AST time to within 12 h [46]. They found that when bacteria, including *E. coli, S. aureus,* and *Pseudomonas aeruginosa*, were cultured in blood culture media comprising blood (0.2 mL) and culture media (0.8 mL), the biofilm formation and bacterial growth could be detected by measuring capacitance changes at f = 0.5 and 10 kHz, respectively. After treated with antibiotics, the sensitivity of bacteria to antibiotics could be judged by this change. This study demonstrated that an aptamer-functionalized sensor could be used as an alternative tool for detecting bacteria and rapid AST in positive blood culture without the need for sub-culturing.

Furthermore, Lee et al. developed an electrical AST (e-AST) system, which shortens the AST time to only 6 h [47]. The e-AST system was composed of 60 aptamer-functionalized capacitance sensors, of which 2 sensors were used for the negative control, 3 sensors for positive control, and other 55 sensors for the determination of antibiotic sensitivity to 11 antibiotics at 5 different concentrations. To evaluate the performance of the e-AST system, 4,554 tests were conducted on 30 clinical strains isolated from septic patients. The results showed an estimated 97% classification agreement between the e-AST system and the gold standard broth microdilution (BMD) test, indicating its great potential for clinical application. Although the diagnosis of sepsis with e-AST may be more expensive, it is significant to save patients suffering from sepsis.

## Aptamer-based detection of sepsis-related biomarkers

In addition to the successful detection of pathogenic bacteria in the early period of sepsis, it is necessary to monitor the biomarkers timely which are helpful to judge the occurrence and stage of sepsis and observe systematic conditions [83]. Biomarkers, especially from the blood, make significant changes of content when inflammatory responses occur in the early time with the happening of two molecular patterns [84]. Changes in their levels indicate a state of inflammatory response in the body that cannot be provided by the methods of diagnosing pathogens. However, it is still a challenge to find an accurate and quantitative way to detect biomarkers in the human blood. Compared with frequently-used methods of detecting biomarkers like mass spectrometry (MS) and antibody-based technologies, aptamer-based sensors have shown a huge potential in recent years because of their superiorities like low costs, wide detection ranges, low immunogenicity, and sufficient sensitivity [85–87]. Several biomarkers are associated with the occurrence and development of sepsis, such as LPS, IL-6, and CRP [88]. In the following sub-section, some aptamer-based sensors for detecting sepsis-related serum biomarkers are discussed.

### LPS

LPS, called endotoxins, are the major glycolipid molecule in the cell walls of gram-negative bacteria which is the main difference between gram-negative and gram-positive bacteria [89]. Many investigations have highlighted the impact of LPS in the development of sepsis and LPS interacts with the special cellular receptors such as Toll-like receptor-4/MD2 and CD14 to produce inflammatory cytokines [90]. The release of LPS can cause a series of cascading inflammatory pathological reactions, which lead to the occurrence of sepsis. Therefore, LPS is considered an important biomarker for the diagnosis of sepsis.

Over the last few decades, the detection methods of LPS have been continuously developed. Previous methods, such as the rabbit pyrogen test (RPT) [91], the Limulus amoebocyte lysate (LAL) [92], the monocyte activation test (MAT) [93], the recombinant factor C (rFC) [94], and the EndoLISA [95], cannot be routinely used to analyze clinical sepsis samples because of their disadvantages of temperature dependence, high cost, inconsistent, and long testing time. However, studies have shown that the LPS test methods based on aptamers were used for the early diagnosis of sepsis in the past few years, which have the advantages of sensitivity, specificity, high affinity, low cost, and time-saving.

**Fig. 8:**
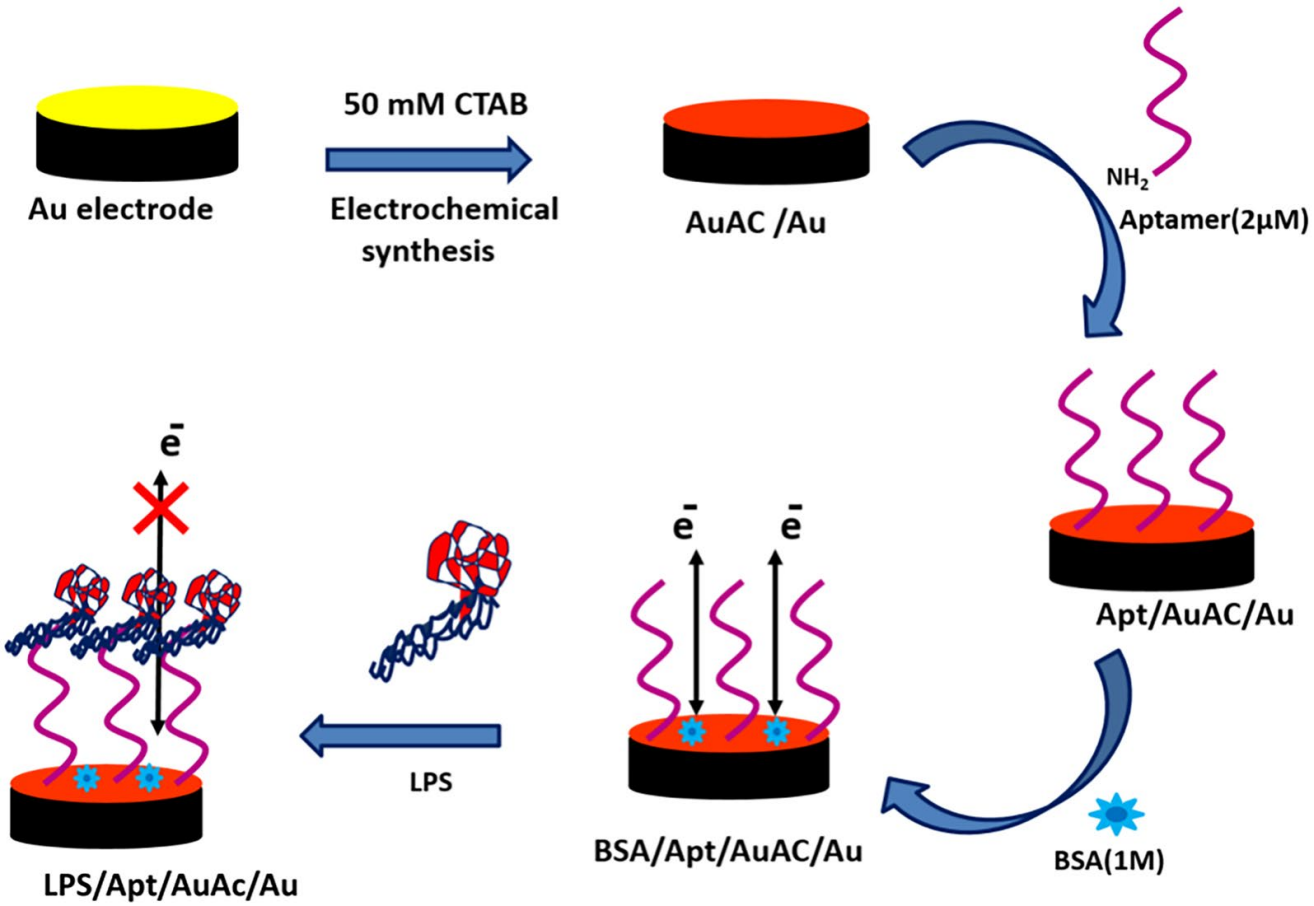
Schematic diagram of the preparation and working principle of Apt/AuAC/Au sensor [52]

To capture LPS, electrodes have been modified by graphene oxide (GO) and GNPs to detect LPS. Kim et al. used a method based on the nonequilibrium capillary electrophoresis of equilibrium mixtures to identify LPS [50]. It did not include the systematic evolution of ligands by exponential enrichment (NECEEM-based non-SELEX) method. This method constructed an electrochemical aptamer sensor on a gold electrode with a high-affinity LPS binder B2. The linear detection range of the electrochemical aptamer sensor for LPS was from 0.01 to 1 ng/mL. Compared with the traditional strategy of diagnosing sepsis, the time of this method was significantly shorter, however, the extensive application of this aptamer sensor was limited by its low sensitivity. To improve their sensitivity, signal amplification strategies should be considered. Bai et al. designed a new type of aptamer-based electrochemical platform through the combination of two typical signal amplification strategies to achieve the ultra-sensitive detection of LPS [51]. Firstly, the three-way DNA junction-aided enzymatic recycling could increase the electrical signal by increasing the number of capture probes. Besides, the GO nanocomposite material further enhanced the electrochemical signal. The sensitivity of this method was down to femtogram level (8.7 fg/mL), with a linear range of 6 orders of magnitude (from 10 fg/mL to 50 ng/mL). This method obtained better sensitivity compared with the previous study, but the complex manufacturing process of the aptamer sensor would limit its clinical application. Interestingly, Posha and co-workers reported an ultrasensitive electrochemical biosensor that does not require additional signal amplification strategies [52]. Gold clusters were used as electrodes because of their excellent merits, such as fast electron transfer and good water solubility, and then the strong affinity of 5' end amino groups of the aptamer was fixed on the surface of the gold electrode. An electrochemical biosensor could be produced to monitor the concentration of LPS by using the allowed or blocked electron transfer in the surface-assembled molecule. Aptamers immobilized by gold atomic cluster were mediated in this biosensor. The lower detection limit of this sensor was 7.94 × 10^–21^ M, which was at the attomolar level. And the linear range was from 0.01 aM to 1 pM, which was with a linear response of 9 orders of magnitude (Fig. 8). Therefore, this aptamer biosensor had higher sensitivity, simple structure and preparation process, and had a broad application prospect in the detection of LPS in sepsis.

**Fig. 9:**
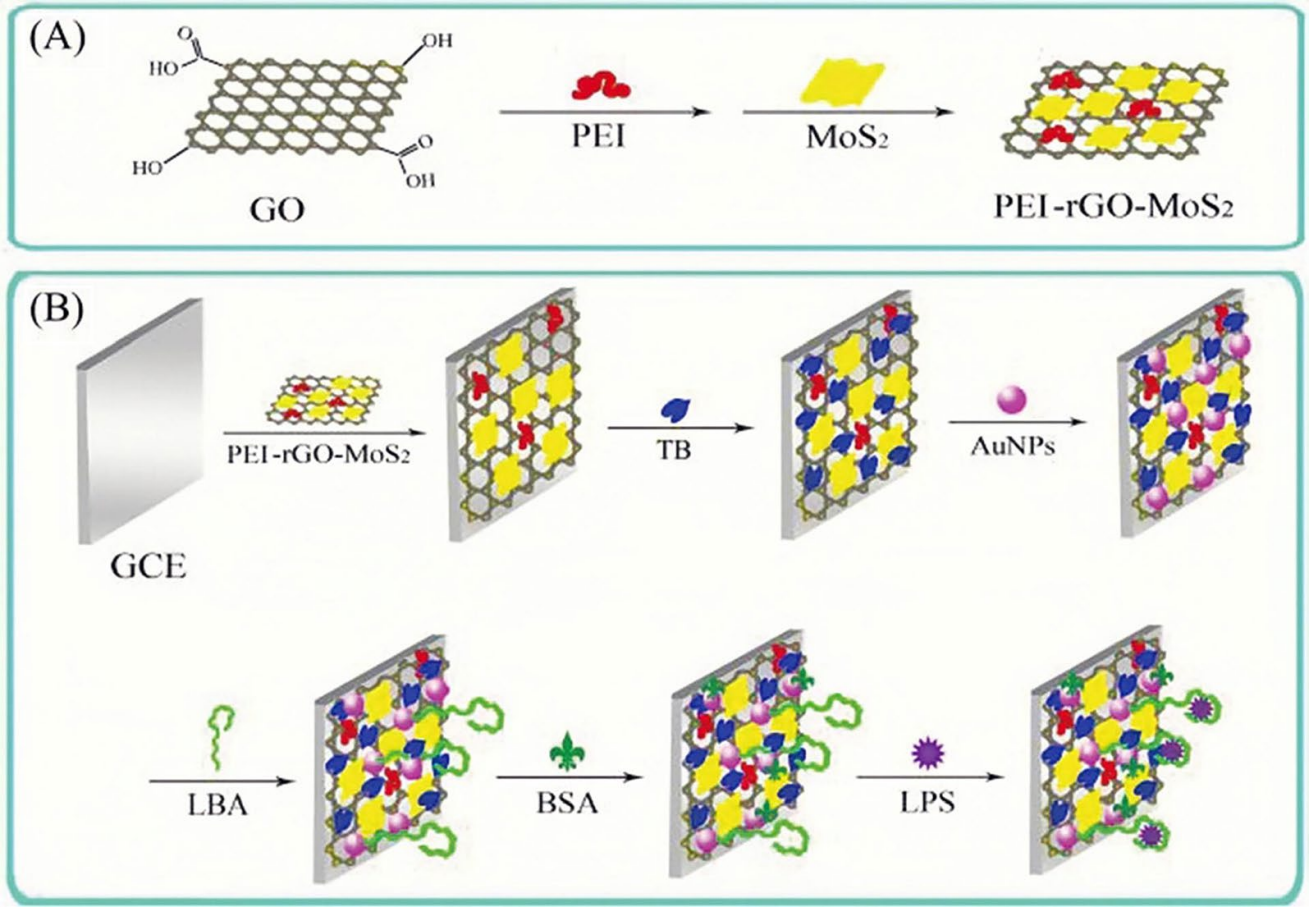
**A** Schematic diagram of the fabrication of PEI-rGO-MoS2. **B** Schematic representation of the coating of the aptamer sensor [55]

Besides, in the last few years, RGO and AuNPs have been used to immobilize aptamers. Compared with GO, RGO has higher electrical conductivity, better ductility, thermal stability, and has received more attention in the biological analysis [96]. For example, Pourmadadi et al. modified aptamers on the surface of a glassy carbon electrode (GCE) via RGO and AuNPs, which were successfully used in the analysis of serum of patients and normal people [53]. In another case, the research by Yazdian et al*.* used RGO-Au NPs to modify electrodes to immobilize thiolated aptamer that specifically binds to endotoxin [54]. After using aptamers immobilized by RGO, the nanomaterial with better performance enabled the aptamer sensor to possess higher sensitivity in LPS detection. The detection lower limit and dynamic range of the sensor were 0.2 fg/mL and 0.001–0.01 pg/mL, respectively. Further, molybdenum disulfide (MoS2) was also applied as the matrix of the biosensor with the application of RGO and AuNPs (Fig. 9) [55]. The high electrical conductivity and large specific surface area of the new nanocarrier can greatly amplify the electrochemical signal and enhance the sensitivity of the aptamer sensor. It was linear in the range of 5.0 × 10^−5^ to 2.0 × 10^–2^ ng/mL, and the lower LOD was 3.01 × 10^−5^ ng/mL. In addition, the method had a good recovery rate for serum samples and a broad application prospect in the field of trace analysis of LPS in sepsis diagnosis. The label-free aptamer sensor in this study could simplify the operation sequence and had a fast response speed. Some studies by An et al. and Ji et al. also used label-free aptamer sensors to detect LPS [56, 57].

**Fig. 10:**
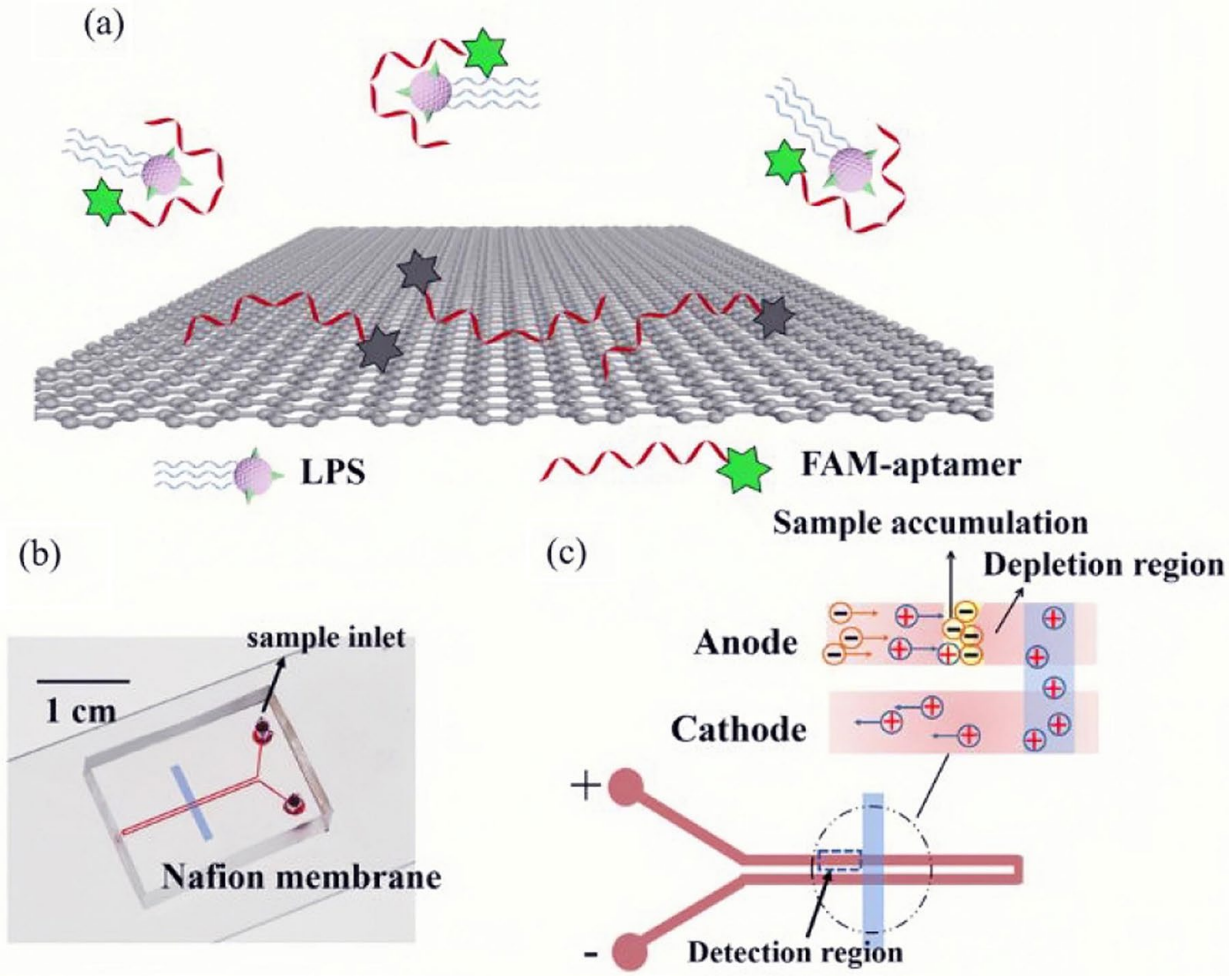
The interaction principle of determination of LPS by coupling FAM-aptamer and rGO on a microfluidic biochip. **a** Schematic diagram of the fluorescence formation of LPS; **b** Schematic representation of the PDMS microfluidic CI-ES-chip; **c** Voltage scheme applied for the LPS preconcentration and the CI-ES mechanism [59]

Also, there were some studies using aptamers labeled with 6-carboxyfluorescein (6-FAM) to detect LPS. For instance, Zhang et al. designed a 6-FAM labeled aptamer as a fluorescent probe to detect LPS, by combining the advantages of the aptamer's specific binding ability and the fluorescence quenching effect of GO [58]. However, the linear range of this probe for LPS was 25–1600 ng/mL and the lower LOD was 15.7 ng/mL. Therefore, the detection sensitivity was low, and the study did not mention whether this method could be effectively applied in clinical practice. There was a significant improvement in Niu et al. [59]. Based on the fluorescence quenching effect in the study, they designed a microfluidic chip based on the continuous injection-electrostacking to couple RGO and FAM-aptamer so that the aptamer could be combined with LPS to achieve the purpose of detection. The detection limit of this method was 8.3 fM (8.3 × 10^–4^ Eu/mL) and the sensitivity is higher. This aptamer-based biosensor can detect LPS in injections and serum of human and sepsis model mice, and can quickly distinguish between gram-negative bacteria from gram-positive bacteria and fungus (Fig. 10). Taken together, this method is simple, sensitive and specific, and has a good correlation with the gold-standard LAL assay. As a practical application, it can be used for the detection of sepsis in the clinic.

### IL-6

IL-6 is a cytokine that takes part in many immune and inflammatory responses [97]. It plays an important role in the process of the hepatic protein during acute inflammation[98]. In addition, the high level of the IL-6 is a symbol of the high risk of death especially in intra-abdominal sepsis [99]. Compared with a low concentration of IL-6 in the normal condition (lower than 10 pg/mL), the level of IL-6 rises rapidly (even more than 1 pg/ml) when sepsis occurs in adults [100]. Therefore, detecting the change of IL-6 concentration is of great significance for the early diagnosis of sepsis.

To detect IL-6 rapidly, aptamers are usually combined with NPs, such as AuNPs, graphene, nanotubes, to construct sensor systems.

**Fig. 11:**
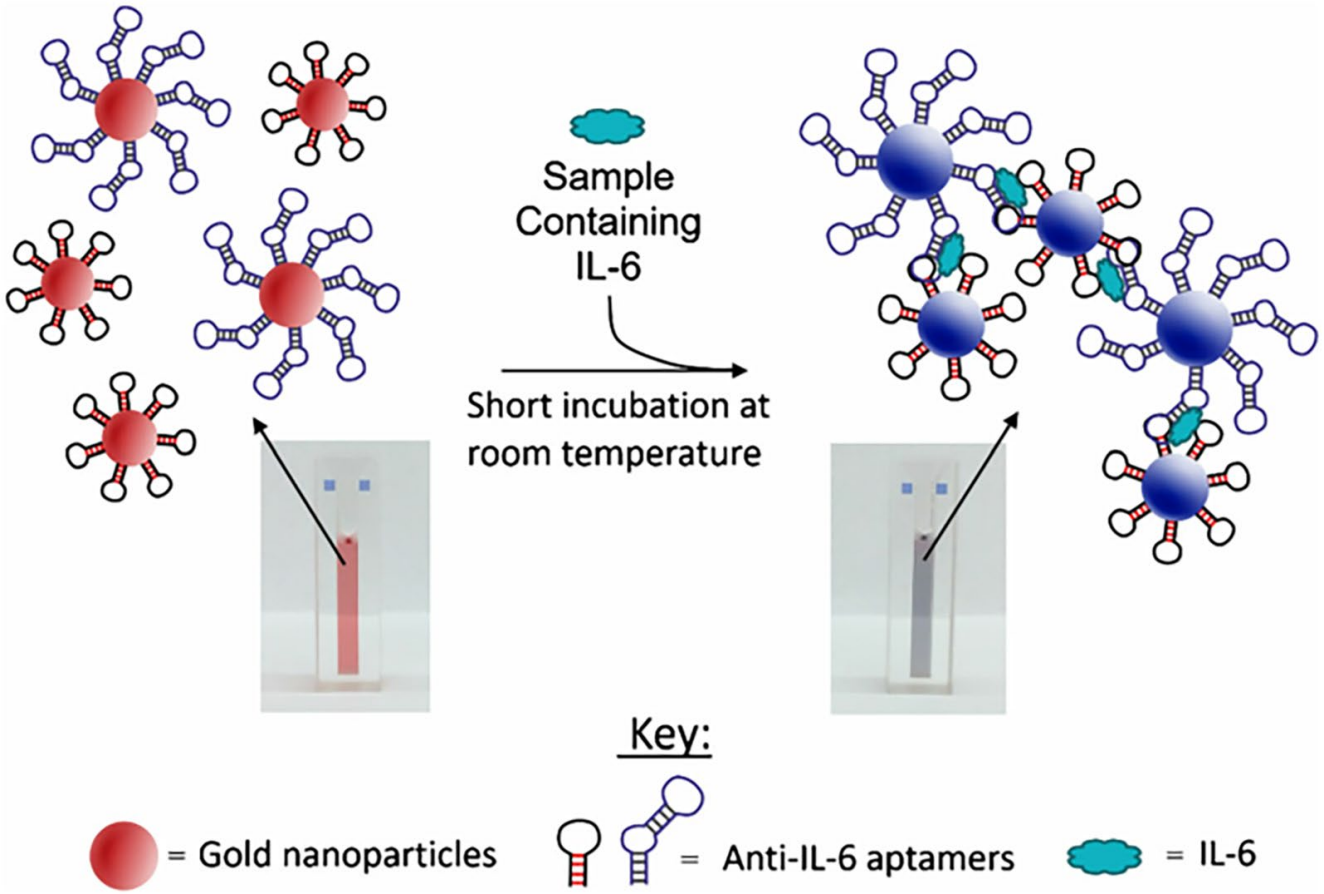
The process of the sandwich-style and color changes [60]

For instance, to develop an optical sensor, Giorgi-Coll et al. combined many aptamers with gold nanoclusters, which was called “sandwich-style” (Fig. 11) [60]. When aptamers recognized and attached IL-6, the gold nanoclusters were aggregated and made the color of the solution change from red to pink in few minutes, which could be measured by a spectrophotometer or a plate reader. It provided a fast method for detecting the concentration of IL-6 in human serum albumin by an optical sensor, which contributes to diagnosing sepsis quickly in clinical.

Tertis et al*.* developed an electrochemical sensor that was made up of glassy carbon electrodes coated with GNPs and aptamers fixed via gold-sulfur bonds [61]. The designed sensors could test the concentration of IL-6 from 5 pg/mL to 100 ng/mL with a detection limit of 1.6 pg/mL. The sensor could be re-used and be applied to detect other biomarkers in the clinic, which was helpful to diagnose sepsis.

For another sensor like microfluidic sensor, Khosravi et al*.* modified aptamers conjugated 1-Pyrenebutanoic Acid Succinimidyl Ester (PASE) on the nanotube biosensors [62]. The conductance was reduced with the increase of IL-6 concentration, which caused the change of the electrical signal. This was the first time to use the PASE conjugated aptamers to detect IL-6 and the strategy not only achieved label-free techniques but also completed 10 pg/mL sensitivity in serum.

Hao et al*.* combined the aptamer with a graphene-based field-effect transistor (GFET) to build the aptameric graphene-based based field-effect transistor(A-GFET) [63]. By relying on online signal processing circuits, the detection of IL-6 was carried out in ten minutes with the limit of 140 fM. In addition, the transistor could detect IL-6 which was stored for a long time. After that, they improved the A-GFET by using PASE as a linker, which caused the range of detection was extended to 618 fM and the sensing performance of A-GFET was improved [64].

### CRP

CRP is an acute-phase protein produced by the liver, which is a common biomarker for the diagnosis of infection and inflammation in clinical treatment. The levels of CRP are elevated after infection and inflammation [101]. The level of CRP mostly depends on the amount of tissue damage currently occurring, and it can rise 1000-fold after infection or tissue damage within 24 to 48 h [102]. Elevated serum CRP concentration indicates a potential risk of organ failure and sepsis [103]. Besides, CRP has been widely used as biomarker of neonatal sepsis [104]. Therefore, detecting CRP is of great significance for the early diagnosis of sepsis.

Previous detection techniques, such as antibody-based tests [105] and enzyme-linked immunosorbent assay (ELISA) [106], have been proven to successfully detect CRP, however, some limitations are present. For example, antibody activity is not stable, and different animals have various immune responses, which leads to different specificity and sensitivity to CRP. ELISA can also cause inaccurate results by the color and composition of the medium used in assays [107].

**Fig. 12:**
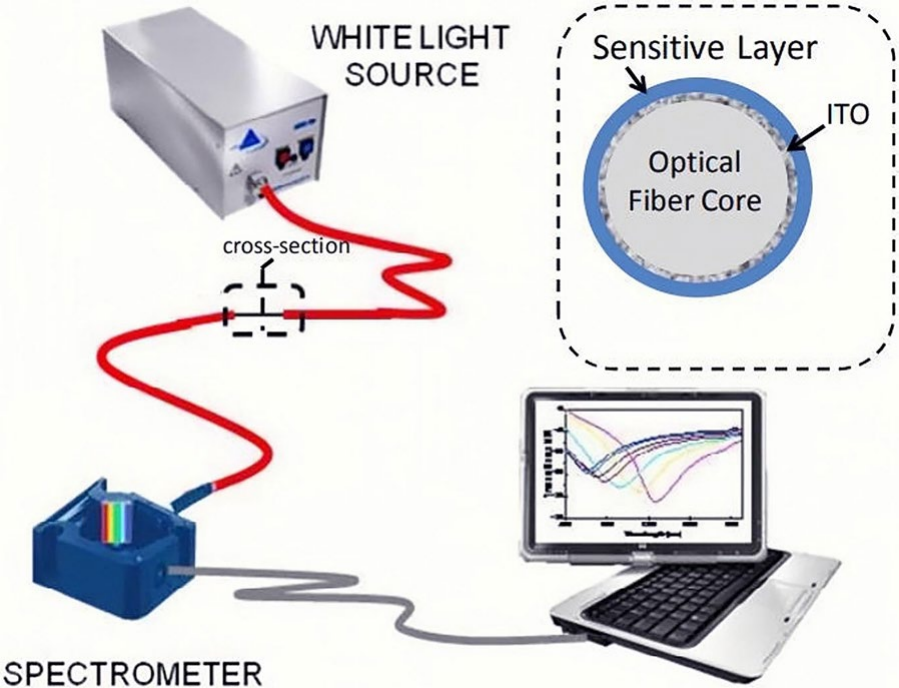
The experimental setup of a fiber optic biosensor [65]

Recently, aptamers have been applied in the detection of CRP. With the rapid development of optical fiber technology, the research of optical fiber sensors (OFS) received increasing attention. Generally, OFS works by detecting changes in light propagation caused by external stimuli. Compared with traditional sensor technologies, OFS is resistant to electromagnetic interference and high temperatures [108]. In addition, OFS has already been used to monitor pH, respiration rate, heart rate, and body temperature in the biomedical fields [109, 110]. Zamarreño and co-workers prepared a biosensor for rapid response and real-time monitoring of sepsis [65]. In this assay, researchers used a layer-by-layer (LbL) technique to combine CRP aptamer film with a fiber optic refractometer based on lossy mode resonances (LMRs). As aptamers bound to CRP, the refractive index of the sensitive coating changed (Fig. 12). The developed sensor could measure CRP in the range of 2–20 mg/L in less than 15 min. This experiment provided a new direction of diagnosis for early sepsis.

Besides, Luminex xMAP technology for clinical diagnosis is a hot topic in the field of bioscience. It allows simultaneous detection of multiple analytes, using a lower sample size and shorter culture time. Furthermore, multiple types of aptamers can be incorporated into this technology [111]. Porschewski et al*.* reported the use of aptamers in the xMAP technology [112]. To expand the application of aptamers, Bernard et al*.* coupled an RNA aptamer that binds CRP to beads to act as the trapping agent [66]. Biotinylated anti-CRP antibody coupled to fluorescently labeled streptavidin was used to quantify CRP. An assay for the detection of CRP was successfully established with the detection limit of 0.4 mg/L in diluted serum.

**Fig. 13:**
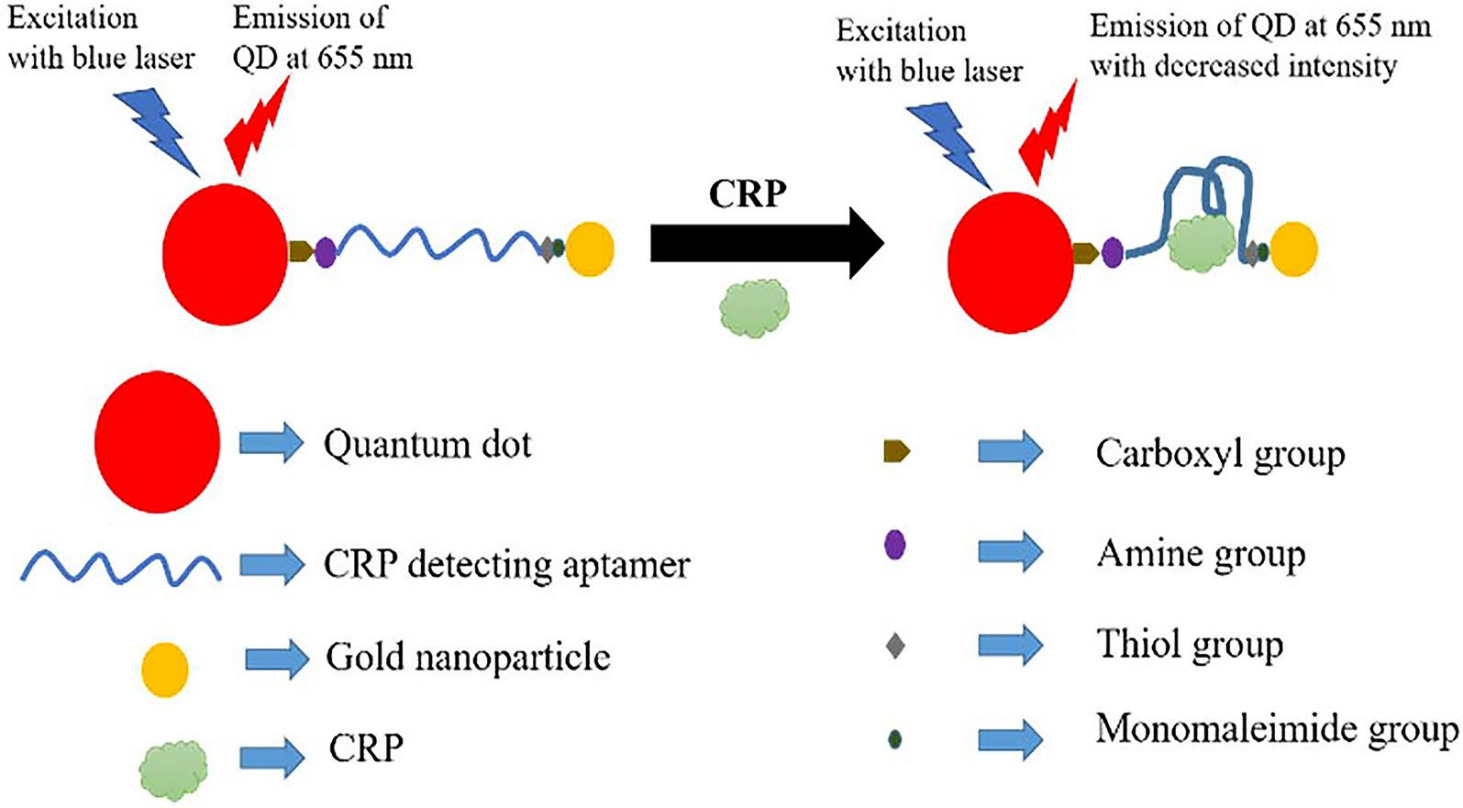
A decrease in the photoluminescence of the nanosensor when CRP binds to the DNA aptamer [67]

In the last few years, NPs have been used to detect CRP in sepsis. Ghosh et al*.* reported an optical nanosensor using DNA aptamer as the main sensing element [67]. This biosensor combined a deoxyribonucleic acid aptamer with a quantum dot on the 5’ terminus and a GNPs on the 3’ terminus. When the aptamer bound to CRP, the photoluminescence intensity decreased based on the principle of fluorescence resonance energy transfer (FRET) (Fig. 13). The nanosensor was highly specific for CRP and the minimum detection limit was 1.77 pM. This detection system was synthesized by the wet chemical method and was simple in design. By using specific DNA aptamers, it could also be applied to detect other molecules.

CRP is also a generic biomarker for inflammation, cancer, cardiovascular, and neurological diseases. Currently, several aptamer-based detection strategies have been constructed, e.g., surface plasmon resonance [113], electrochemical [114], Photoelectrochemical (PEC) [115], etc.

## Conclusions and perspectives

Diagnosing sepsis at the early stage is of great significance, which could guide doctors to use effective symptomatic and antibiotic treatment. Aptamer-based biosensors may be a powerful complement to the traditional diagnostic method "blood culture" because of their accessibility, rapidity, and stability. The review summarized the recent progress of aptamer-based biosensors in the detection of bacteria and biomarkers for the diagnosis of sepsis. It is highly expected that innovative aptamer-based applications will emerge through rational design and development to achieve an excellent clinical performance.

Although some preliminary success has been achieved in the area of sepsis diagnosis by taking advantage of aptamer-based nano-biosensing systems, some challenges still remain for advancing this technology.

First, many aptamers have been selected against the sepsis-related targets, but only a tiny minority of aptamers’ properties have been investigated. The safety of most aptamers has not been demonstrated, which limits their biological/clinical applications. In addition, the aptamer secondary and tertiary structures can be easily affected by temperature. To maintain its spatial configuration, the aptamers used for sepsis diagnosis should be selected according to the temperature at the time of clinical testing. Cold storage may also change their optimal folded conformation, which leads to lower detection accuracy. Thus, it is very important to develop a technique to avoid such potential thermally-induced conformational issues. Besides, the binding performance of aptamers may be affected in complex matrices. Therefore, it is necessary to keep aptamers' normal function in working matrices, such as using chemical modifications to enhance their stability, and testing the performance of aptamer-based biosensor in its specific working environment is also needed.

Second, although numerous work has been reported for proof-of-concept, there is still no available kits that can be applied at clinical or industrial level. Developing more efficient platforms, which may remedy the cost and inconvenience of aptamer-based nano-biosensing systems, will accelerate the translation from the bench to the clinic. Therefore, aptamer-integrated high-throughput analysis platforms could offer an ideal strategy to detect multiple pathogens and biomarkers from human biofluids that are commonly involved in the occurrence and development of clinical sepsis.

Third, it is critical to have the ability to design an aptamer-based nano-biosensor to offer ultra-high sensitivity and reproducibility with a large dynamic range simultaneously, because the signals of the sensor could be interfered from the biological molecules and aptamer are susceptible to degradation in biological media. A promising strategy may be the appropriate chemical modification in aptamer-based nanosensors, e.g., using polyethylene glycol to enhance resistance to exonucleases.

Fourth, it was reported that some specific aptamers have potential applications in the treatment of sepsis. Integration of the diagnosis and treatment in sepsis could be a promising strategy in the future research direction.

Overall, the aptamer-based sensor systems have a great potential for the early diagnosis of sepsis due to their excellent merits, such as high sensitivity, fast detection speed, wide detection range, easy mass production, etc. The specificity of this type of detecting technique makes it possible to detect different targets only by changing the type of aptamers. In addition to detect pathogens and biomarkers, it can monitor the real-time growth of bacteria and biofilms and also conduct drug sensitivity tests. With the continuous development of interdisciplinary research, it is predictable that the aptamer sensor system will become a crucial diagnostic tool for sepsis.

## Data Availability

Without restrictions.
